# Disentangling adaptive evolutionary radiations and the role of diet in promoting diversification on islands

**DOI:** 10.1038/srep29803

**Published:** 2016-07-13

**Authors:** Daniel DeMiguel

**Affiliations:** 1 Institut Català de Paleontologia Miquel Crusafont, Universitat Autònoma de Barcelona, Edifici Z, C/de les Columnes s/n, Campus de la UAB, 08193 Cerdanyola del Vallès, Barcelona, Spain

## Abstract

Although the initial formulation of modern concepts of adaptive radiation arose from consideration of the fossil data, rigorous attempts to identify this phenomenon in the fossil record are largely uncommon. Here I focus on direct evidence of the diet (through tooth-wear patterns) and ecologically-relevant traits of one of the most renowned fossil vertebrates-the Miocene ruminant *Hoplitomeryx* from the island of Gargano-to deepen our understanding of the most likely causal forces under which adaptive radiations emerge on islands. Results show how accelerated accumulation of species and early-bursts of ecological diversification occur after invading an island, and provide insights on the interplay between diet and demographic (population-density), ecological (competition/food requirements) and abiotic (climate-instability) factors, identified as drivers of adaptive diversification. A pronounced event of overpopulation and a phase of aridity determined most of the rate and magnitude of radiation, and pushed species to expand diets from soft-leafy foods to tougher-harder items. Unexpectedly, results show that herbivorous mammals are restricted to browsing habits on small-islands, even if bursts of ecological diversification and dietary divergence occur. This study deepens our understanding of the mechanisms promoting adaptive radiations, and forces us to reevaluate the role of diet in the origins and evolution of islands mammals.

Islands have long been recognised as nature’s test tubes of great value in studying macroevolutionary processes even since Darwin’s early proposal of natural selection^1^. This lies in the fact that islands involve the emergence of novel and unexplored ecological opportunities for immigrant lineages to appear and proliferate^2^. The initial colonists, encountering new and untapped resources and lacking ecological competitors and predators, often radiate in novel and heterogeneous habitats more rapidly than in the mainland^3^. This evolutionary idiosyncrasy of islands is characterized by an unbalanced accumulation of newly formed species—with unusual morphological and/or physiological adaptations^4^,^5^,^6^—through which unoccupied ecological space is filled by a burst in ecological diversification *in situ* rather than colonization^7^.

Recently, much progress has been made in understanding the timing and pattern of this important outcome of the process of evolution^8^, referred to as adaptive radiation, which has been shown to be as the main cause of the great diversification of ecological and morphological traits in a rapidly speciating group of organisms on islands^2^. To date, the majority of adaptive radiation studies are biased towards bird species from oceanic islands (interesting in this regard are the Galapagos finches, Hawaiian honeycreepers and lobeliads, the Gulf of Guinea white-eyes, the Australian corvoids or Madagascan vangids, and a plethora of others^9^,^10^,^11^,^12^,^13^,^14^), mostly because they have occurred very recently and are readily accessible to scrutiny. However, we know relatively little about terrestrial—especially mammal—species to explain why some lineages undergo adaptive radiation and others do not^14^,^15^,^16^,^17^; and is unclear how important adaptive radiation is over temporal scales that span large portions of the history of life^18^. Under this view, the fossil record provides striking case studies for a fuller understanding of the rates and patterns of phenotypic change within mammalian clades on islands, and can add a new dimension to the study of adaptive radiations. Although the initial formulation of modern concepts of adaptive radiation arose from consideration of the fossil data, rigorous attempts to identify adaptive radiation in the fossil record are still uncommon^18^.

The latest Miocene record of the Gargano palaeo-island, in Central Mediterranean (Fig. 1), is among the most renowned in the world, as it records the occurrence of unique unbalanced biotas with manifest signs of rapid insular adaptation from different sites^19^,^20^—usually only one or a few fossil sites are known from a certain island, but in Gargano c. 75 localities are known and represent sequential time slices^21^. This insular evolution is particularly perplexing in the case of *Hoplitomeryx*^19^, which literally means armed-ruminant, and shows a number of anatomical features that successive authors^19^,^20^,^22^,^23^ have qualified as “unique”, the most striking one being the presence of five cranial appendages, never seen before in any other—extinct or presently known—mammal (Fig. 2A). Since its discovery, *Hoplitomeryx* has been the subject of great controversy and debate (see methods). On the one hand, the development of a number of unique anatomical features obscures its phylogenetic relationships within the Ruminantia^19^,^24^; on the other hand, we know little about the time and mode of colonization of Gargano by *Hoplitomeryx*’s ancestors^25^,^26^,^27^,^28^. What we know, however, is that the special abiotic conditions of Gargano seem to have been an ideal scenario for rapid adaptive divergence and perhaps also permitted more rapid occupation of newly available and novel niche space by this enigmatic mammal. Indeed, the island has been characterized by several examples of prodigious diversification events, in particular among the micromammal fauna^29^ (Fig. 2B).

Despite to receiving a substantial amount of attention, there has been little effort to assess the evolution and palaeobiology of *Hoplitomeryx* and, surprisingly, no work has focused on its dietary capabilities. For any animal, diet is the most direct connection with its environment and so, key for our understanding of the evolutionary processes under which it radiates^30^. Thus, in a resource-limited and small palaeo-island such as Gargano, where *Hoplitomeryx (i)* can be observed from its beginnings, *(ii)* isolated rapidly, *(iii)* documents an unusual diversity of forms and *(iv)* persisted for long periods (over millions of years) of time, it is no surprise that diet had surely driven selection forces and mechanisms that are responsible for its adaptability, behavioural ecology and evolution. *Hoplitomeryx* emerges, therefore, as one of the most promising, but poorly known, models of fossil vertebrates to investigate causes and trajectories of evolutionary radiations on islands and understand processes at the nexus between evolution and ecology.

This research relies therefore on the initial working hypothesis that *Hoplitomeryx*, the only large mammal species on Gargano, shows signs of rapid early proliferation of phylogenetic and ecological diversity after invading the island that, with the proper methodology, can be estimated to illustrate how island mammals diversified in novel directions more explosively and rapidly than in the mainland. To do so, I propose a different approach which ultimately aims to test from a palaeodietary viewpoint the most likely causal forces under which adaptive radiations emerge on small islands and the intrinsic capacity of species to evolve rapidly in the face of posible climatic variability, by focusing on the tooth wear and ecologically relevant phenotypic (body mass and molar crown height) traits of the species of *Hoplitomeryx*.

## Results

Results of the tooth wear, hypsodonty and body mass estimations are reported in Table 1A. The values of each molar cusp shape (MCS) mean and occlusal relief (OR) (expressed as percentages), as well as the average mesowear score (MS), for each *Hoplitomeryx* species are shown.

### Long-term patterns of tooth wear

All the species of *Hoplitomeryx* show occlusal enamel surfaces with a predominance of sharp cusps and high oclussal relief, while there is a considerable variation in the proportion of rounded cusps depending on the species. *Hoplitomeryx* sp. 1 and *Hoplitomeryx* sp. 4 have more rounded apices than the remaining species. Fossil taxa do not show any incidence of blunt cusps or, with the exception of *Hoplitomeryx* sp. 2 (represented by the largest sample), low relief (Table 1A). All these facts presumably exclude a high abundance of abrasives in the diets^31^, such as phytolith-rich grasses and exogenous dust and grit. The scores recorded cover a narrow range of levels in food abrasiveness, ranging from 0 in *Hoplitomeryx* sp. 3 (similar to *Alces alces* and *Rhinoceros sondiacus*; scores converted from data of Fortelius and Solounias^31^), 0.1 in *Hoplitomeryx* sp. 2 and *Hoplitomeryx* sp. 4 (*Odocoileus virginianus* and *Okapia johnstoni*), to 0.2 in *Hoplitomeryx* sp. 1 (*Dicerorhinus sumatraensis*). This also indicates low levels of abrasion, as typical of leaf browsers, and points to a somewhat larger amount of abrasive material in *Hoplitomeryx* sp. 2 than in the remaining species. Mesowear signals reveal, therefore, attrition dominated wear surfaces and low levels of dietary abrasion, compatible with the signal characteristic of extant browsing mammals^31^. In addition, results are not compatible with the inclusion of external abrasives (dust, grit or soil) being habitually incorporated into the diet by *Hoplitomeryx* species in the natural environments of Gargano.

### Body mass estimation

Four body size classes are present in *Hoplitomeryx*, for which mass ranges from approximately 10 kg in *Hoplitomeryx* sp. 1 (similar to the largest species of *Deinogalerix* from Gargano^6^) to 50 kg in *Hoplitomeryx* sp. 4. *Hoplitomeryx* sp. 2 and *Hoplitomeryx* sp. 3 are intermediate-sized species with 21 and 30 kg, respectively (Table 1A). One of the two intermediate size classes (that of *Hoplitomeryx* sp. 2) is by far the most abundant in Gargano, and is found in all the fissures in which this species occurr with the rest of the fauna. Kruskal-Wallis analyses (*Hoplitomeryx* sp. 2, H = 2.695 and *p* = 0.4411; and *Hoplitomeryx* sp. 4, H = 0.5026 and *p* = 0.77781) and ANOVA (*Hoplitomeryx* sp. 2, F = 0.9043 and *p* = 0.453; and *Hoplitomeryx* sp. 4, F = 0.2441 and *p* = 0.7885) of the most abundant species failed to find significant size differences between the specimens of the same species according to biozones. If compared to living browsing ruminants^32^, *Hoplitomeryx* species are comparable in increased order of size to the common duiker *Sylvicapra grimmia* (13 kg), the black-backed duiker *Cephalophus dorsalis* (20 kg), the dibatag *Ammodorcas clarkei* (31 kg), and the gerenuk *Litocranius walleri* (45 kg). Another case of similar taxonomic and size variability to that found in *Hoplitomeryx* is the endemic Pleistocene Cretan deer *Candiacervus*, which is represented by eight morphotypes distributed over six size classes^33^, and body mass estimated between about 20 kg to 315 kg^22^. Apparently, it has a broader range size than *Hoplitomeyx*, but it is intersting to note the presence of a considerable large species in both *Hoplitomeyx* and *Candiacervus*.

### Molar crown height analysis

*Hoplitomeryx* sp. 1 and *Hoplitomeryx* sp. 2 are mesodont forms according to their hypsodonty index (HI = 0.81 and 0.83, respectively), though they are in the lower threshold of this morphological condition, while *Hoplitomeryx* sp. 3 and *Hoplitomeryx* sp. 4 are classified as brachydont (HI = 0.70 for both) (Table 1A). Despite the fact that hypsodonty entails a certain degree of phylogenetic signal^34^, it is generally accepted (though there are some inconsistencies in this respect^35^) that low indices (i.e., usually a brachydont condition) reflect species with low dietary abrasion (i.e., leaf/fruit-dominated browsers) in relatively dust/grit-free habitats, and higher indices (i.e., hypsodont taxa) usually indicate greater dietary abrasion (i.e., grass-dominated feeders) and more open, arid environments^34^. It is apparent from the values obtained that all the species of *Hoplitomeryx* experienced a low degree of dietary abrasion and inhabited closed (no dry) areas in Gargano, hereby confirming findings from tooth wear patterns.

### Multivariate analyses

A first explorative cluster analysis based on mesowear variables (Fig. 3A) yields two main clusters separating browser (cluster A) from mixed feeder and grazer taxa (cluster B). All *Hoplitomeryx* species cluster in A with extant leaf browsers. *Hoplitomeryx* sp. 2 and *Hoplitomeryx* sp. 3 are grouped with *A. alces, A. americana, O. virginianus* and *D. bicornis* in subcluster A1. *Hoplitomeryx* sp. 1 and *Hoplitomeryx* sp. 4 are in turn included in subcluster A2, together with the remaining extant browsers and the mixed feeder *A. marsupialis*, which are characterized by higher incidences of rounded cusps. Overall, the analyses indicate a folivorus component for all the fossil species of *Hoplitomeryx*. None of the species clusters with extant mixed feeders or grazers. The canonical variate analysis (CVA) (Fig. 3B, Table 1B, and Supplementary Tables S2 and S3) confirms that the investigated mesowear variables provide a satisfactory dietary discrimination, with 77.8% of extant taxa correctly classified, and 68.9% in cross-validation. CV1 separates mixed feeders and grazers (positive values) from browsers (negative values) mostly on the basis of the MCS, whereas CV2, more influenced by OR, does not enable as a clear distinction among dietary categories than CV1. The discriminant analysis (Table 1B) based on the CVA classifies all the *Hoplitomeryx* species as browsers (Fig. 3B).

## Discussion

Tooth mesowear patterns of *Hoplitomeryx* traced through time offer for the first time evidence on the relationship of diet and ecological diversification in insular mammals, and provide direct evidence of response to several events that affected its continuous evolution in Gargano. *Hoplitomeryx* species primarily browsed on forbs and dicots and inhabited considerably closed areas, as reflected by the low mesowear scores and the predominance of cusp sharpness and high relief of the teeth. Species show no signs of hypsodont dentitions, and there are no large differences in this trait among lineages. Despite this, not all the species are equal in their degree of hypsodonty, as the smallest forms are found to be slightly more hypsodont than the largest ones, with *Hoplitomeryx* sp. 1 and *Hoplitomeryx* sp. 2 considered as mesodont and *Hoplitomeryx* sp. 3 and *Hoplitomeryx* sp. 4 being brachydont. It may be added that most of the body mass values reported are lower than those for highly dimorphic ungulates^36^, thereby supporting the view^20^ that *Hoplitomeryx* size groups cannot be explained by sexual dimorphism and constitute instead different species. Regarding temporal differences, the different sizes are not neatly distributed over the biozones, as there are two or more size groups per fissure and biozone (except the youngest one). Accordingly, differences in foraging strategies (i.e., diet composition) and body size may have allowed *Hoplitomeryx* species to coexist on the same range in Gargano.

### The evolution of diet in relation to species and ecological diversity: causes and trajectories of the adaptive radiation

Despite a general leafy browsing behaviour for the species of *Hoplitomeryx*, with mesowear scores that vary from 0 to 0.5 (i.e., a range of abrasion that is still quite narrow in the sense that it represents low abrasion overall for most of the species—when compared to extant ungulates^31^), a chronological order of the data (Fig. 4A) shows a somewhat asymmetrical pattern and a statistically significant fluctuation in diet composition. That is, there is a dietary shift, though all of the taxa clearly have an attritive (less abrasion) diet and stay in the browsing realm. Although not all biozones are equally represented by the fossil material, the following evolutionary changes are supported by the samples that yielded the richest and most reliable information.

A first important phenomenon is the exceptional species diversity of *Hoplitomeryx* even since its first record, with the occurrence of three different forms in the oldest localities of Gargano (Supplementary Table S1) and scores that range from 0 to 0.5, this latter being the highest score recorded among the fossil sample (Fig. 4A). Such differences reveal the existence of differential resource utilization and niche segregation for the various species studied, with *Hoplitomeryx* sp.2 and *Hoplitomeryx* sp.3 being more dependent on soft foods and *Hoplitomeryx* sp.4 already having a comparatively higher proportion of tough abrasive elements (similar to the browse-dominated mixed feeder *T. imberbis*^31^) or incorporating some exogenous grit into its diet. Although only a few specimens have been found in biozone 1, this pattern reveals that *Hoplitomeryx* did not undergo uniform diversification dynamics after invading Gargano, but instead a prodigious adaptive radiation characterized by an accelerated accumulation of species, concomitant with an early burst of phenotypic and ecological diversity.

The first co-occurrence of all *Hoplitomeryx* species is registered in biozone 2 (Fig. 4A), which corroborates that the genus diversified rapidly. It is worth noting, however, that the ecological diversity of the species starts to decline at this time, as denoted by a decrease of the wear scores and their restriction to a rather narrower range of diet composition. This trend culminates in biozone 3 (e.g., Chiro 5A and Chiro 27), in where all species occurred with very low scores (MS = 0) in a soft-leafy browsing niche. It is important to stress that all these species exhibited a higher level of abrasion in biozone 2. Among extant ungulates, there are only browser species with scores lower than 0.2, which indicates a very attritive diet probably based on leaves and twigs for the species in biozone 2 and 3 which did not contribute to dietary abrasion in any appreciable way, with scarce or even no abrasive foods selected. The high abundance of very soft food items in this stage apparently allowed selective foraging in all the species to consume not the entire plant but the most succulent and nutritious (i.e., high energy-yielding) plant parts (e.g., selecting leaves over stem, or selecting the shrub with the larger leaves or thickest twigs, etc)^37^. One implication of this is that *Hoplitomeryx* would undergo a rapid increase in population density that, in tandem with the lack of natural predators and food competitors in Gargano^19^, might have caused a decline in mortality rates and a phase of overpopulation in biozone 3. As observed in small islands^4^,^5^,^38^,^39^, such a lack of control of the population growth rate has led to a degradation of the preferred vegetation in Gargano and increased intraspecific competition for these food resources.

A trend towards increased mesowear values followed by a new burst in ecological diversification is observed after biozone 3, as reflected especially by the small-sized *Hoplitomeryx* sp. 1 and *Hoplitomeryx* sp. 2 (MS = 0.4 and 0.2, respectively) in biozone 4. This trend is further expressed in biozone 5, with all the species here recorded (e.g., in Chiro 20A, Chiro 20E, Gervasio, San Giovannino, etc) having scores of around 0.35. Although species stayed with browse, all this indicates higher levels of abrasion control^40^ in the tooth wear equilibrium of *Hoplitomeryx*, with a preference for more abrasive foods and/or higher grit exposure after biozone 3, and species most likely occupying somewhat more open areas. There might be three different phenomena to explain this greater amount of tooth wear. First, a greater reliance on tubers and roots, which would involve higher tooth abrasion by species as a consequence of digging into the ground to reach them. Second, and because numerous extant mixed feeders (e.g., *T. oryx, A. marsupialis* and *T. imberbis*, this latter being an attrition-dominated mixed feeder) show similar mesowear scores to those of the *Hoplitomeryx* species in biozone 5, another possible and probably interrelated explanation is that some grasses and abrasive elements (probably including more grit encroachment on foods) became a part of the diet of these species, at least on a seasonal basis. Lastly, extensive frugivory could also account for the higher scores of the species, as ruminants involved in fruit-eating show more rounded and less sharpened cusp apices than leafy-browsers as a result of tip-crushing wear associated with hard fruit coverings and seed coats and/or soil adhering to fallen fruits^31^. This latter, however, may not be the case to explain the increase in dietary abrasion during the period represented by biozones 4 and 5, as none of the *Hoplitomeryx* species fell in the range of fruit-browsers in a new CVA (Fig. 3C) that includes extant representatives (especially duikers) of this feeding type. Given that dental mesowear is best thought of as an abrasion (and not a dietary) index, though it indeed provides a glimpse of the dietary capabilities of the species, the ability of dental microwear to indicate subtle, short-term (e.g., seasonal) variations in diet^41^ could be used for teasing out the determinants of the dietary shift (i.e., silica phytoliths of plants vs. grit/dust, or a shift that combines all the aforementioned reasons) and implement the results obtained here. At the same time, two possible explanations, not neccessrily mutually exclusive, may shed light on this new dietary breadth expansion (i.e., wider ecological divergence) in *Hoplitomeryx* after biozone 3: *(i)* an adaptive strategy to survive—that is, species of *Hoplitomeryx* were probably forced to expand the range of consumable food items (with higher abrasiveness and lower nutritional value) in an attempt to exploit vacant ecological niches and ensure the survival of the group; *(ii)* a response to a rapid climatic and environmental instability—hence, aphase of aridification could have put *Hoplitomeryx* in contact with novel plant species (e.g., more abrasive items such as grasses and other monocots, dicots rich in phytoliths, grit loaded foliage, etc) and thus increased the likelihood of occupation of niches through ecological fitting. Although it may seem quite surprising that an arid episode may have led to some grass being available, grasses (and related plants) are indeed the most common type of food eaten by living ruminants in desert and arid areas^40^, while forbs, shrubs and other ligneous vegetation are the least used forage resource. This alternative implies therefore that an abiotic (external) factor caused its ecological range to expand.

Finally, and although only one sample is existent in biozone 6—and this is certainly not due to limitations related to sampling because species normally present in all biozones are lacking in this last interval^22^—, much lower levels of dietary abrasion and a return to a soft-leafy browsing diet are seen, as denoted by the very low scores (MS = 0) of *Hoplitomeryx* sp. 2.

The rates and trajectories of body size diversification are also modeled (Fig. 4B), showing that ecological diversification rates occur without significant change in body size, although slightly smaller body size is observed to occur with a pulse of increased dietary abrasion (from biozone 4 to 5) in some species. Thus, results show that *Hoplitomeryx* sp. 2. and *Hoplitomeryx* sp. 4 are 10.5% and 7% smaller, respectively, than their preceding relatives.

### Environmental change and co-evolution of *Hoplitomeryx* with micromammals

Changes in the feeding spectrum here detected through the dental mesowear of *Hoplitomeryx* perfectly match changes of the whole small-mammal community, also easily affected by climate instability—although less than ruminants^42^. Figure 4C combines the evolution in diet of *Hoplitomeryx* (through the representation of the wear pattern of its most widespread *Hoplitomeryx* sp. 2) with the main changes in the micromammal association of Gargano. A first evidence of the existence of a phase of aridification that intervened in the continuous insular evolution of Gargano is the significant evolutionary change undergone by the murid *Mikrotia*. The record of this highly ubiquitous genus is characterized by an abundance of well-preserved material, representing several (at least five) species/lineages that exhibit a high degree of evolutionary differentiation^43^. Small-sized and less derived *Mikrotia* species are widespread in the most ancient fissures, whereas larger-sized and morphologically derived lineages occurred in the youngest ones. The first appearance of the largest *Mikrotia (M. magna*) lineage in latest biozone 3 (Chiro 27) coincides with dietary homogeneity in *Hoplitomeryx* (Fig. 4C). Changes through time in *Mikrotia* include a very marked growth in size, development of propalinal chewing, increasing hypsodonty, and an increase in size and complexity of m1 and M3^44^,^45^. These macroscopic changes, accompanied by a tendency to thicken the enamel wall of molars^46^, appear to be an adaptation to a very abrasive diet driven by climatic deterioration^43^,^47^. Thus, the most morphologically derived teeth of *Mikrotia* belong to specimens from San Giovannino (biozone 5)^44^,^45^, and reflect a diet that included grasses and the ingestion of dust and grit as a consequence of new environmental conditions^43^. The most derived *Mikrotia* populations coincide therefore with the maximum dietary abrasion reached by *Hoplitomeryx*. Besides findings from *Mikrotia*, a marked trend towards aridification on Gargano archipelago has been also invoked through the evolutionary pattern found in the lagomorph *Prolagus* (a very distant relative of extant pikas)^44^, and the disappareance in biozone 4 of micromammals normally present in all localities, as is the case of the cricetids that cease to exist in the area interval^44^.

Although determining the age of the fissures of Gargano is largely a matter of conjecture^48^, these have been tentatively attributed to a time interval that corresponds approximately to the Tortonian/Messinian^49^. A Late Tortonian (MN11-MN12) or Messinian (MN12-MN13) age represents therefore the best fit for the time of this event of intensified aridification in Gargano and the shift towards a somewhat increased dietary abrasion in *Hoplitomeryx*. From a wider perspective, this phase of appearance of new open-land, arid-adapted vegetation types^50^ and decreasing humidity^51^ agrees with the dominating conditions of the Mediterranean in this epoch. This climatic trend culminated with the Messinian salinity crisis (MN 13, 5.96 Ma), which progressively restricted and finally isolated the Mediterranean Sea from the open ocean^52^.

### Evolutionary and ecological implications: island constraints preventing transition among feeding styles

Species of *Hoplitomeryx* appear to have been sensitive to demographic (high population density), ecological (competition, few resources and food requirements) and abiotic (climate) drivers in Gargano. This variety of causes, probably acting in combination, pushed species to a phase of expansion in diet breadth (i.e., expanding from a soft-leafy to a more abrasive-dominated browsing) preceding strong phenotypic change (e.g., acquisition of extremely hypsodont molar teeth, loss of teeth, evergrowing incisors, shortened premolar series, etc, as recognized in other Mediterranean island ruminants^53^,^54^) to escape from overpopulation. Much of the divergence in diet took place during a phase of aridification that favoured the expansion of *Hoplitomeryx* species into vacant or novel niches.

Although a number of additional factors not investigated (such as adjustments in morphology/physiology, geological changes leading to the appearance of novel environments, etc) might influence diversity, diet emerges as paramount in determining ecological diversification on small and resource-limited islands, and represents a density-dependent variable explaining much of the rate and magnitude of insular radiations. It is important to stress, however, that such a dietary expansion in the species did not lead to an immediate change in their major feeding (browsing) type and so, species were not involved in prominent grass-eating. On continents, where mammals adapt more slowly^55^,^56^, resources are not limited in variety and extent^57^ and the diversification dynamics act differently^58^, the expanded use of different foods among species of *Hoplitomeryx* may have easily represented the initiation towards a dietary specialization, probably through an initial transition to a more varied diet through a mixed feeding type (i.e., mixture of both browse and grasses), more in accordance with the new environmental circumstances (increased aridity, seasonality and openness of the landscapes) of the epoch. This view is supported by the fact that generalist—both recent and extinct—species are known to better adapt to climatic instability and changing environments than specialized ones^40^.

The following hypothesis needs to be further tested (and the present study implemented through dental microwear in order to offer more specificity and better resolution of the results), but the model here presented strongly supports the view that, despite the potential to exhibit multiple changes in diet composition, the capacity of ruminants to undergo changes in the feeding style on small islands is potentially low. Interestingly, although many of the extinct insular ruminants may have showed a shift to a more divergent dietary ecology to be better suited for life in a variety of habitats, the constraints imposed by small-sized islands might have been more serious, and prevented evolutionary transition from browsing to mixed feeding or grazing. To interpret this, it is necessary to understand what ruminants can eat and why they do, and to consider the environmental conditions and the specific selection pressures^5^ under which taxa like *Hoplitomeryx* occur. The type of feeding developed by a ruminant is strongly dependent on the quality and quantity of the forage available^37^. This is because grasses and their plant parts (such as stems and twigs) are generally of lower nutritional quality than browse (leaves and fruits)^59^,^60^,^61^. In order to meet their nutritional requirements, mixed feeder and grazer species require larger quantities of food than do browsers^62^. The consequence of this is that the limiting food resources on small islands, such as Gargano, aggravated by the effects of overpopulation seem to prevent the acquisition of mixed and grazing diets among mammals. This hypothesis is congruent with recent findings for other endemic herbivorous clades from the fossil record^63^. Clearly, this constrain may have played an important role in the origins, diversification and evolution of a broad range of island mammals, both recent and extinct, such as elephants, hippos, bovids and deer.

In conclusion, this study provides a detailed picture of the adaptive radiation undergone by *Hoplitomeryx* that is drawn from an innovative approach combining long-term patterns of tooth wear with ecologically relevant traits. Adaptive radiation in *Hoplitomeryx* resulted from ecological opportunity. Demographic, ecological and abiotic factors are recogized as primary drivers of the evolution and ecological diversity of species in Gargano. A pronounced event of overpopulation and a rapid phase of increased aridity determined the rate and magnitude of radiation, and pushed species to expand their diets from soft-leafy to more abrasive-dominated browsing. Results show for the first time that herbivorous mammals are highly restricted to browsing habits on small islands, even if bursts of ecological diversification and divergence in diet occur. Finally, this study highlights that a wide range of research questions can benefit greatly by incorporating data from the fossil record. This is especially important for an accurate prediction of ecological shifts (exploitation of vacant ecological niches, species interactions, etc) and species diversification on islands in the face of current and future climatic variability.

## Methods

### The insular fauna of Gargano and the case of *Hoplitomeryx*

**Figure 1:**
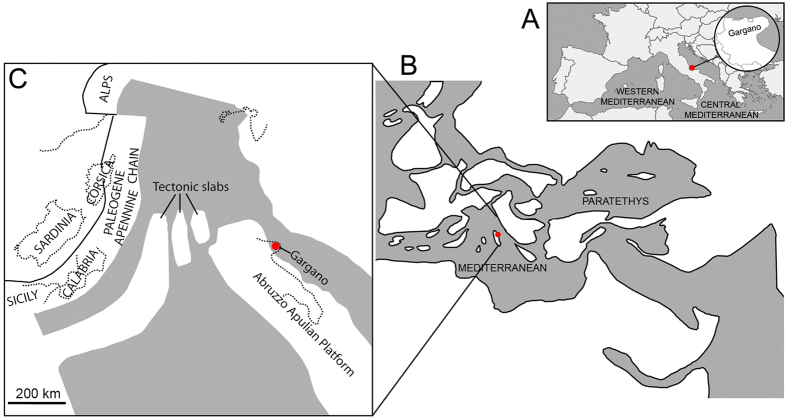
Schematic maps showing the palaeoisland of Gargano. (**A**) Geographical setting of the present-day Peninsula of Gargano, part of mainland Southern Italy from the Early Pleistocene onwards but an island from the Late Miocene and Early Pliocene. (**B**) Reconstruction of the palaeogeography of the peri-Mediterranean and peri-Paratethyan areas. (**C**) Magnified view of the Central Mediterranean and the position of Gargano in the Abruzzo-Apulian Platform. Red dots show the position of Gargano. Maps reproduced under permission from elsewhere^25^: Mazza, P.P.A. Hoplitomerycidae (Ruminantia, Late Miocene, Central-Southeastern Italy): whom and where from? Geobios 2013, 46:511-520. Copryright © 2013 Elsevier Masson SAS. All right reserved.

**Figure 2:**
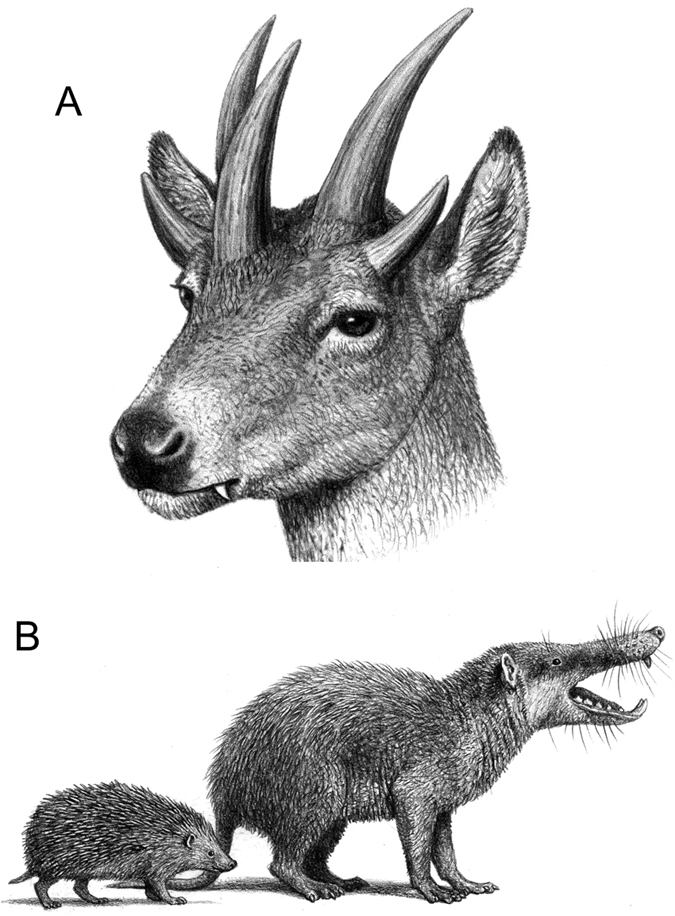
*Hoplitomeryx* and the insular fauna of Gargano. (**A**) Life reconstruction of *Hoplitomeryx* showing the presence of five cranial appendages (two paired postorbital ones, and an unpaired appendage projecting between the eyes on the caudal part of the nasals) and large, flaring sabre-like upper canines. (**B**) Example of the highly endemized association of Gargano, with a reconstructed life appearance of the giant hedgehog *Deinogalerix* (in scale with modern *Erinaceus europaeus*). Artwork by Mauricio Antón.

**Figure 3:**
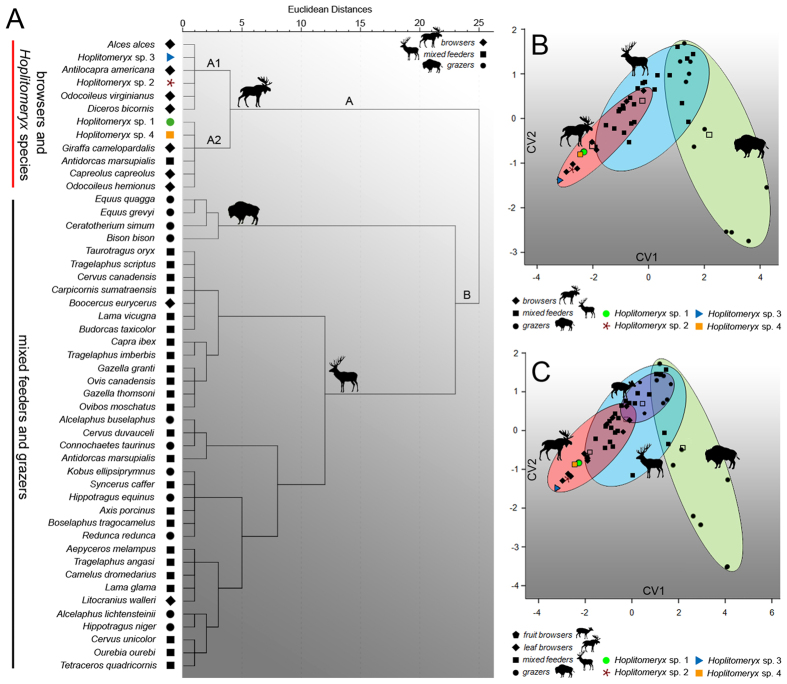
Statistical mesowear grouping showing the relative placement of *Hoplitomeryx* species. (**A**) Hierarchical cluster based on the percentage of high occlusal relief, round cusps and blunt cusps. Data of extant species from^31^. (**B**) Results of the canonical variate analyesbased on three dietary categories and mesowear (high relief, round and blunt cusps) variables. (**C**) Results of the canonical variate analyes based on four dietary categories (including frugivorous species) and the same mesowear variables. The figure was designed through the combined use of SPSS Statistics 19, Adobe Illustrator CS6 and Adobe Photoshop CS3 software.

**Figure 4:**
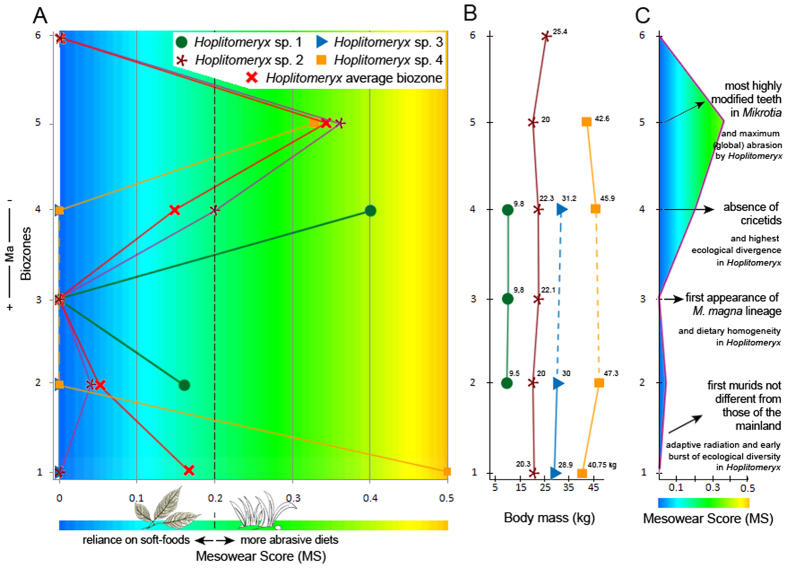
Causes and trajectories of adaptive radiations. (**A**) Evolutionary history of the diet of *Hoplitomeryx* species traced according to chronological sequence of the biozones. (**B**) Evolution of body size through time. (**C**) Evolution of the wear pattern of the most widespread species and correlation with main changes in the micromammal association. The figure was designed through Adobe Photoshop CS3 software.

The Mediterranean is an area of intense tectonic activity, leading to dramatic changes in the palaeogeography throughout all the Cenozoic. One of the most active orogenetic zones during the Tertiary was Italy, in where islands emerged and submerged repeatedly and mammal faunas from that region testified such a phenomenon^21^. The most important Italian island faunas were discovered in the 1970s, and belong to the fossils from fissure fillings on Gargano. The material from this island, now firmly joined to the Italian mainland, was retrieved from the Late Miocene karstic fissures fillings in quarries between Apricena and Poggio Imperale (Province of Foggia, Southern Italy)^19^ (Fig. 1). Apart from the ruminant *Hoplitomeryx*, the bulk of the assemblage, often referred to as the *Mikrotia* fauna—because the particular abundance of this giant murid^48^—is composed of a full range of other giant rodents (the dormouse *Stertomys* and the hamster *Hattomys*), the giant erinaceid *Deinogalerix* (Fig. 2B), crocodiles, chelonians, amphibians and birds^29^,^64^,^65^,^66^. Practically all mammals of the Gargano show extraordinary morphological signs of insularity^20^ such as, among others, the presence of aberrant and giant forms among the micromammals^29^.

Despite its long history of scientific study, palaeontologists and evolutionary biologists consider *Hoplitomeryx* as one of the most controversial topics of current interest. Some of this disputation focuses on the number of genera and species erected, with some authors^23^ considering 1 (type) genus and 6 species (*Hoplitomeryx matthei* [type species]*, H. apruthiensis, H. apulicus, H. falcidens, H. magnus*, and *H. minutus*) while others^22^ willing to accept as many as 2 genera (*Hoplitomeryx* and *Scontronmeryx*) and 10 species (*Hoplitomeryx matthei* [type species], *H. devosi, H. macpheei* and *H. kriegsmani*, and *Scontronmeryx minutus* [type species], *S. apruthiensis, S. apulicus, S. falcidens, S. magnus* and *S. mazzai*) from the same fossil material. As the mainland ancestors of *Hoplitomeryx* are unknown, another focus of controversy concerns its phylogenetic and—still unresolved—link within Ruminantia. By showing two lacrimal orifices and closed metatarsal gulleys, it was originally accommodated in the Cervoidea^19^. *Hoplitomeryx*, however, does not possess antlers, the most characteristic feature of cervids, as its cranial appendages are unpaired, non-deciduous and unbranched. As a consequence, a more recent overview of its systematic position^24^ provided findings for linking it as either between antilocaprids and bovids, or antilocaprids and giraffids, since a supposed ancient origin (in the Late Oligocene or Early Miocene) failed to accomodate it in any of the advanced ruminant lineages. The last matter of conjecture is when and how the *Hoplitomeryx* ancestors reached the palaeo-island. A mode of colonization by land-bridges that connected Gargano with the Balkans across the Adriatic Sea in the Late Oligocene is embraced by some authors^25^,^26^, while sweepstake dispersals at the end of the Late Miocene are considered by others^27^,^28^.

### Dataset

The material studied consists of teeth of *Hoplitomeryx* from the paleo-island of Gargano. The following 4 species have been recognized on the basis of evident differences in size and certain morphological variation of teeth (Supplementary Fig. S1), without formally naming them—as there is difficulty to reliably assign morphotypes to any of the *Hoplitomeryx* spp. because of the lack of detailed studies comprising upper dentition. It has been thus recognized in increased order of size: *Hoplitomeryx* sp. 1, *Hoplitomeryx* sp. 2, *Hoplitomeryx* sp. 3, and *Hoplitomeryx* sp. 4. The material considered for analysis belongs to 28 different localities, distributed in 6 different biozones (Supplementary Table S1)^44^,^65^,^66^. All specimens are housed at the Naturalis Biodiversity Center, Leiden (the Netherlands).

### Long-term patterns of tooth wear

To evaluate whether and how *Hoplitomeryx* species resulted in explosive evolutionary radiation and flourished in a variety of ecological settings, the tooth-based mesowear method^31^ of dietary evaluation—one of the most powerful tools for documenting dietary abrasion in fossil species^40^,^67^—was used. A total of 87 upper molars (belonging to 87 different individuals) were analyzed, and individual molar cusp shape (MCS) and occlusal relief (OR) scores were converted into a single mesowear score (MS) for each fossil species following the five-point scoring system proposed by Rivals *et al*.^68^.

### Body mass

Body mass^69^ is usually predicted through equations based on proximal limb bone measurements^70^ and cranial^71^ or dental^32^ measurements. In order to asses correlation between dietary evolution and phenotypical and ecological diversity within lineages^69^, an estimation of the size of *Hoplitomeryx* species based upon upper (preferably second) molar length^32^ was utilized.

### Molar crown height

Tooth height (i.e., hypsodonty) is one of the most important morphological traits influencing evolutionary and ecological processes^72^, and typically found on islands^73^. Molar crown height analyses were thus applied as a measure of the total dietary abrasion of the items ingested by *Hoplitomeryx* species.

A more comprehensive description of the Gargano fauna and *Hoplitomeryx*, dataset, paleodietary inference and statistical techniques is available in the Supplementary Information.

## Additional Information

**How to cite this article**: DeMiguel, D. Disentangling adaptive evolutionary radiations and the role of diet in promoting diversification on islands. *Sci. Rep.* **6**, 29803; doi: 10.1038/srep29803 (2016).

## Supplementary Material

Supplementary Information

## Figures and Tables

**Table 1:** (A) Chronological record of the *Hoplitomeryx* species, and summary of body mass, hypsodonty and dental mesowear, and (B) results of the discriminant analysis (and predicted group) according to the mesowear features.

Species	Zone	A											B				
		BM		HYP		Mesowear							Discriminant Analysis				
		N	kg	N	HI	N	pS	pR	pB	pH	pL	MS	1st PG	p	D^2^	2nd PG	D^2^
*Hoplitomeryx* sp. 1	2, 3, 4	7	10.5	2	0.813	9	80	20	0	100	0	0.2	B	0.945	0.114	MF	5.752
*Hoplitomeryx* sp. 2	1, 2, 3, 4, 5, 6	34	20.7	13	0.832	48	91.02	8.98	0	97.7	2.3	0.153	B	0.674	0.789	MF	8.691
*Hoplitomeryx* sp. 3	1, 2, 4	7	30.1	3	0.703	12	100	0	0	100	0	0	B	0.373	1.971	MF	12.021
*Hoplitomeryx* sp. 4	1, 2, 4, 5	15	48.2	5	0.701	18	86.2	17.8	0	100	0	0.137	B	0.903	0.205	MF	6.329

Abbreviations: body mass (BM); number of specimens measured (N); kilograms (kg); hypsodonty (HYP); hypsodonty index (HI); percentage of specimens with sharp (pS), rounded (pR) and blunt (pB) cusps; percentage of specimens with high (pH) and low (pL) occlusal relief; mesowear score (MS); predicted group (PG); Squared Mahalanobis distance (D^2^); browsers (B); and mixed feeders (MF). See Table S2 and S3 for further details on the discriminant analysis.
