# Supplementary material for: Disentangling adaptive evolutionary radiations and the role of diet in promoting diversification on islands
Source: Sci Rep. 2016 Jul 13;6:29803. doi: 10.1038/srep29803 (PMC4942836; doi:10.1038/srep29803)
Supplement: Supplementary Information [file srep29803-s1.pdf]

Supporting Information for

Disentangling adaptive evolutionary radiations and the role of diet in promoting diversification on islands

Daniel DeMiguel

Contents

Supporting Methods..........1

Dataset..........1

Long-term patterns of tooth wear..........1

Body mass..........2

Molar crown height..........2

Statistical tests..........3

Supplementary Figure..........3

Supplementary Tables..........4

References..........7

Supplementary Methods

Dataset

As a consequence of a high incongruence between the taxonomic classifications proposed to date to accommodate species (1, 2), and given that the vast majority of the dental (especially upper) elements have not been taken into account for systematic purposes, none of the current classifications for *Hoplitomeryx* has been adopted for this study. Because the material belongs to fissure fillings, the absolute age of the localities is not at all clear. *Hoplitomeryx* sp. 2 is the species with more abundant sample and largest chronological distribution (supplementary Table S1).

Long-term patterns of tooth wear

Dental mesowear reflects the cumulative wear imposed on teeth during a relatively long period (months, years) of feeding activity as a consequence of the abrasive

elements intrinsic to the plants themselves, and also exogenous grit encroaching on food items (3). All available isolated upper molars and tooth rows containing dental specimens were sampled. Although both the lower and upper dentition of *Hoplitomeryx* has been studied, only the upper one is here considered for analysis following (3, 4). It must be also stressed that the lower dentition shows a higher variability in size than the upper one, with probably 5 or 6 or species considering the lower teeth, while 4 ones according to the upper. Unworn, juvenile/senescent specimens and teeth with poor preservation of occlusal enamel surfaces or taphonomic alterations were discarded from analysis, following Fortelius and Solounias (3). In the original formulation of the method (3), cusp apices were classified by two variables, molar cusp shape (MCS; sharp, rounded or blunt, according to the degree of facet development) and occlusal relief (OR; high or low, depending on the relative difference in height between the tip of the cusp and the intercusp valley). However, MCS and OR are not independent categories, since higher relief cusps tend to be sharper than low relief cusps, and cusps with no relief are always blunt. Therefore, a mesowear score (MS) was here computed for each fossil species (that is, individual MCS and OR scores were converted to a single scoring) according to the five-point scoring system proposed by Rivals et al. (5) as follows: a score 0 is given to teeth with a combination of high relief and sharp cusps; 1 to teeth with high relief and rounded cusps; 2 to teeth with low relief and rounded cusps; 2.5 to teeth with low relief and sharp cusps; and 3 to teeth with low relief and blunt cusps. The fact of treating mesowear as a univariate scoring allows for a rapid and easy way to represent dietary data as a single data point. For comparative purposes, the mesowear scores for extant species were converted from original data of Fortelius and Solounias (3). As a baseline sample, a set of 45 extant ungulate (artiodactyl and perissodactyl) species (and 1764 specimens) with well-known diets compiled by Fortelius and Solounias (3) was used for comparative purpose. The dataset was partitioned into browser (N=9), mixed feeder (N=25) and grazer (N=11) species.

Body mass

Body size is fundamental for nearly every aspect of organismal function and important underlaying the diversity of feeding niches in ruminants (6). In general, large ruminants must feed on diets of low quality because they require large quantities of food, and the more abundant plants and plant parts (e.g., stems or twigs) are generally of

lower nutritional quality than less abundant, higher-quality parts (e.g., forbs, leaves or fruits) (6). Only adult specimens, in which the length growth is complete, were considered for analysis. Results were compared to body mass estimations obtained for other endemic ruminants (7).

Molar crown height

Hypsodonty is fundamentally an adaptive response to increasing demands for wear tolerance and functional durability as a consequence of the development of more abrasives in a progressively more open and dry-adapted vegetation (8). Accordingly, it yields information about feeding ecology and habitat structure (especially aridity degree) (9). The mean hypsodonty value was calculated for each *Hoplitomeryx* species by averaging ordinated scores. Measurements were taken on unworn molars or those least affected by wear. The values of the height to length ratio of the upper (second, if available) molars were determined with the acronym HI (Hypsodonty Index), and teeth classified as brachydont (with a ratio of less than 0.8), mesodont (ratio of 0.8-1.2) and hypsodont (a ratio > 1.2) (8).

Statistical tests

Hierarchical, complete-linkage (Ward´s method) cluster analyses based on Euclidean distances (Fig. 2A), and discriminant Canonical Variate Analyses (CVA) (Fig. 2B and C, and supplementary Tables S2, S3) were performed to characterize dietary traits of *Hoplitomeryx* spp. following (4). The tests were developed using the combination of rounded and blunt cusps, and high relief as criterion variables. Cluster analysis was intended to explore the similarities in mesowear patterns between extant ungulates and *Hoplitomeryx* species. CVA, in turn, was intended to evaluate the reliability of these mesowear variables for distinguishing between the various dietary categories defined for extant taxa, as well as to classify fossils to these categories. Extant ungulate species were thus included *a priori* in one of the three dietary categories described above, whereas the extinct taxa were left unclassified and classified *a posteriori* on the basis of the classification probabilities derived by the analysis from Mahalanobis squared distances to extant group centroids. Kruskal-Wallis nonparametric tests also were performed for body size to determine whether sample medians were significantly different from each other. Between group differences were evaluated by *one-way*

analysis of variance (*ANOVA*). Descriptive and inferential statistics were computed using SPSS Statistics 19 software.

Supplementary Figure

Supplementary Figure S1. Results of the CVA based on two metric variables. Analysis based on molar length and width of the sample of *Hoplitomeryx* from Gargano showing species differentiation in 4 different types (symbols and colors of *Hoplitomeryx* species in a as in Fig. 3). Specimens not suitable for metrical study were discarded of the CVA.

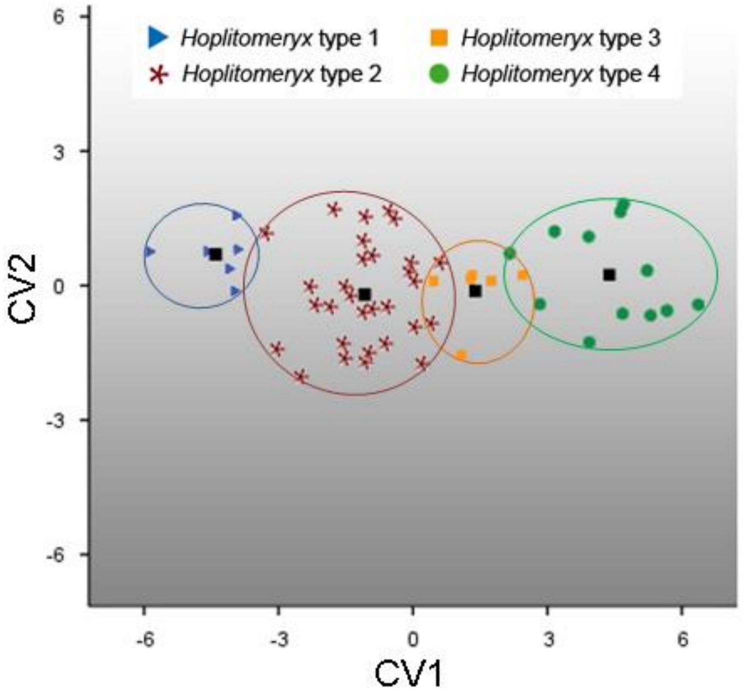

Supplementary Tables

Supplementary Table S1. Fissure-fillings (considered as fossil localities) and biozones with dental material of *Hoplitomeryx*.

**Biozones**					
**biozone 1**	**biozone 2**	**biozone 3**	**biozone 4**	**biozone 5**	**biozone 6**
Trefossi 2A	Nazario 4	Chiro 5A	Chiro 2S	Chiro 20A	Pirro 11A

Trefossi 3	Chiro 3	Chiro 27	Chiro 10B	Chiro 20E	
	Chiro 7A		Chiro 10C	Gervasio	
	Chiro 7C		Chiro 10A	Chiro 15	
	Chiro D1		Chiro 14A	San Giovannino	
	Chiro D2		Chiro 14B	San Giovannino 2	
	Chiro D3		Chiro 28A	San Giovannino low	
	Pizzicoli 4		Fina H		
	Pizzicoli 12		Posticchia 1B		

Fissures without dental material of *Hoplitomeryx* are not specified and considered for analysis. Fissures and biozones biostratigraphically dated (with biozone 1 being the oldest) based on the evolutionary stage of the *Mikrotia* lineages (10). Chronological order derives from refs. (11,12).

Supplementary Table S2. Results of the CVA based on mesowear features.

CV	Eigenvalue	Variance (%)	Cumulative variance (%)	Canonical correlation
CV1	2.148	91.0	91.0	0.826
CV2	0.213	9.0	100.0	0.419
**Standardized coefficients of the CV**				
**CV**	**High relief**		**Rounded cusps**	**Blunt cusps**
CV1	-0.746		0.916	0.335
CV2	0.553		0.666	0.060
**Non-standardized coefficients of CV**				
**CV**	**High relief**	**Rounded cusps**	**Blunt cusps**	**Constant**
CV1	-0.035	0.043	0.029	0.288
CV2	0.026	0.032	0.005	-3.967
**Scores for group centroids**				
**CV**	**Browsers**		**Mixed feeders**	**Grazers**
CV1	-2.025		-0.228	2.174
CV2	-0.624		0.393	-0.382

Abbreviations: CV, canonical variate.

Supplementary Table S3. Scores for the two canonical variates in extant and extinct taxa derived by CVA.

Taxon	N	CV1	CV2	Diet
**Extant species**				
Alces alces	30	-3.209	-1.377	BROW
Antilocapra americana	44	-2.578	-1.124	BROW
Boocercus eurycerus	27	-0.795	0.377	BROW
Capreolus capreolus	68	-1.897	-0.675	BROW
Diceros bicornis	34	-2.957	-1.194	BROW
Giraffa camelopardalis	61	-1.860	-0.704	BROW
Litocranius walleri	69	-0.173	0.625	BROW
Odocoileus hemionus	33	-2.026	-0.517	BROW
Odocoileus virginianus	18	-2.726	-1.026	BROW
Alcelaphus buselaphus	76	2.008	-0.240	GRAZ
Alcelaphus lichtensteinii	17	1.341	0.819	GRAZ
Bison bison	15	3.584	-2.746	GRAZ
Ceratotherium simum	26	4.236	-1.545	GRAZ
Connochaetes taurinus	52	1.628	-0.632	GRAZ
Equus quagga	122	2.978	-2.550	GRAZ
Equus grevyi	29	2.787	-2.536	GRAZ
Hippotragus equinus	26	1.495	1.273	GRAZ
Hippotragus niger	20	1.450	0.999	GRAZ
Kobus ellipsiprymnus	22	1.280	1.681	GRAZ
Redunca redunca	77	1.132	1.277	GRAZ
Aepyceros melampus	17	-0.395	0.668	MF
Antidorcas marsupialis	26	-1.899	-0.630	MF
Axis axis	43	1.201	0.342	MF
Axis porcinus	24	1.377	1.341	MF
Boselaphus tragocamelus	15	1.595	1.448	MF
Budorcas taxicolor	38	-0.520	0.321	MF
Camelus dromedarius	16	-0.221	0.795	MF
Capra ibex	24	-1.231	-0.226	MF
Carpicornis sumatraensis	22	-0.903	0.227	MF
Cervus canadensis	19	-0.921	0.286	MF

Cervus duvauceli	50	1.429	-0.084	MF
Cervus unicolor	21	0.761	0.972	MF
Gazella granti	18	-0.615	-0.107	MF
Gazella thomsoni	146	-0.877	-0.319	MF
Lama glama	32	-0.131	0.811	MF
Lama vicugna	12	-0.674	0.466	MF
Ourebia ourebi	128	0.313	0.967	MF
Ovibos moschatus	52	-0.705	-0.532	MF
Ovis canadensis	31	-0.510	-0.082	MF
Syncerus caffer	31	1.044	1.612	MF
Taurotragus oryx	14	-1.035	0.204	MF
Tetraceros quadricornis	21	0.211	0.647	MF
Tragelaphus angasi	20	-0.382	0.678	MF
Tragelaphus imberbis	31	-1.526	-0.154	MF
Tragelaphus scriptus	47	-1.082	0.169	MF
***Hoplitomeryx* species from Gargano**				
Hoplitomeryx sp. 1	9	-2.339	-0.745	
Hoplitomeryx sp. 2	48	-2.738	-1.153	
Hoplitomeryx sp. 3	12	-3.209	-1.377	
Hoplitomeryx sp. 4	18	-2.435	-0.815	

See supplementaryTable S2 for further details on the CVA results.

Abbreviations: N, number of specimens measured; CV, canonical variate; BROW, browsers; MF, mixed-feeders; GRAZ, grazers.

Mean extant species data were taken from ref. (3). See ref. (4) for information regarding the extant dietary categories employed to define groups *a priori*.

References

1. Mazza, P. P. A. & Rustioni, M. Five new species of *Hoplitomeryx* from the Neogene of Abruzzo and Apulia (central and southern Italy) with revision of the genus and of *Hoplitomeryx matthei* Leinders, 1983. *Zool. J. Linnean Soc*. **163**, 1304–1333 (2011).
2. Van der Geer, A. A. E. Systematic revision of the family Hoplitomerycidae Leinders, 1983 (Artiodactyla: Cervoidea), with the description of a new genus and four new species. *Zootaxa* **3847**, 1–32 (2014).

3. Fortelius, M. & Solounias, N. Functional characterization of ungulate molars using the abrasion-attrition wear gradient: a new method for reconstructing paleodiets. *Am. Mus. Novit*. **3301**, 1–35 (2000).
4. DeMiguel, D., Azanza, B. & Morales, J. Trophic flexibility within the oldest Cervidae lineage to persist through the Miocene Climatic Optimum. *Palaeogeogr. Palaeoclimatol. Palaeoecol*. **289**, 81–92 (2010).
5. Rivals, F., Schulz, E. & Kaiser, T. M. Late and middle Pleistocene ungulates dietary diversity in Western Europe indicate variations of Neanderthal paleoenvironments through time and space. *Quat. Sci. Rev.* **28**, 3388–3400 (2009).
6. Clauss, M., Steuer, P., Müller, D. W. H, Codron, D. & Hummel, J. Herbivory and Body Size: Allometries of Diet Quality and Gastrointestinal Physiology, and Implications for Herbivore Ecology and Dinosaur Gigantism. *PLoS ONE* **8**, e68714 (2013).
7. Van der Geer, A. A. E. Systematic revision of the family Hoplitomerycidae Leinders, 1983 (Artiodactyla: Cervoidea), with the description of a new genus and four new species. *Zootaxa* **3847**, 1–32 (2014).
8. Fortelius, M. et al. (2002) Fossil mammals resolve regional patterns of Eurasian climate change over 20 million years. *Evol Ecol Res* 4:1005–1016.
9. Janis, C. M. in *Teeth Revisited: Proceedings* of the *VII International Symposium on Dental Morphology* (eds Russell, D. E., Santoro, J. P. & Sigogneau-Russell, D.) 367–387 (Paris, 1988).
10. Freudenthal, M. Rodent stratigraphy of some Miocene fissure fillings in Gargano (prov. Foggia, Italy). *Scripta Geol*. **37**, 1–23 (1976).
11. Ballmann, P. Fossile Vögel aus dem Neogen der Halbinsel Gargano (Italien). *Scripta Geol.* **17**, 1–75 (1973).
12. Ballmann, P. Fossile Vögel aus dem Neogen der Halbinsel Gargano (Italien), zweiter Teil. *Scripta Geol.* **38**, 1–59 (1976).
